# Complete Genome Sequence of the Novel Giant *Pseudomonas* Phage PaBG

**DOI:** 10.1128/genomeA.00929-13

**Published:** 2014-01-09

**Authors:** Nina N. Sykilinda, Alexander A. Bondar, Anna S. Gorshkova, Lidia P. Kurochkina, Eugene E. Kulikov, Mikhail M. Shneider, Vassily A. Kadykov, Natalia V. Solovjeva, Marsel R. Kabilov, Vadim V. Mesyanzhinov, Valentin V. Vlassov, Valentin V. Drukker, Konstantin A. Miroshnikov

**Affiliations:** Shemyakin-Ovchinnikov Institute of Bioorganic Chemistry RAS, Moscow, Russiaa; Genomics Core Facility, Institute of Chemical Biology and Fundamental Medicine SB RAS, Novosibirsk, Russiab; Limnological Institute SB RAS, Irkutsk, Russiac; Winogradsky Institute of Microbiology RAS, Moscow, Russiad; Novosibirsk State University, Novosibirsk, Russiae

## Abstract

The novel giant *Pseudomonas aeruginosa* bacteriophage PaBG was isolated from a water sample of the ultrafreshwater Lake Baikal. We report the complete genome sequence of this *Myoviridae* bacteriophage, comprising 258,139 bp of double-stranded DNA containing 308 predicted open reading frames.

## GENOME ANNOUNCEMENT

Bacteriophage PaBG (vB_PaeM_BG) was isolated from a sample of lake water and found to infect *Pseudomonas aeruginosa*. Pseudomonads are widely distributed in the ecosystem of Lake Baikal and utilize various compounds as sources of carbon and energy. *P. aeruginosa* is a multidrug-resistant facultative human pathogen that causes hospital-acquired infections and thus is considered to be a serious problem in the medical and veterinary fields. A detailed investigation of phage genomics might help to understand the role of the viruses in ecology and evolution and facilitate the discovery of new therapeutic agents against pathogenic microorganisms.

Bacteriophage PaBG was propagated in the *P. aeruginosa* PAO1 strain. The DNA was extracted (1) from the phage particles concentrated by polyethylene glycol 8000 (PEG8000) precipitation and purified by ultracentrifugation in a cesium chloride gradient (2). The phage genomic DNA library prepared by the Nextera kit (Illumina) was sequenced using the MiSeq next-generation sequencer (300 cycles; Illumina), yielding an average of 75-fold coverage. The genome was *de novo* assembled using the CLC Genomics Workbench (GW) 6.0 software (CLC bio). Terminal sequences and five areas of the phage genome with a higher ratio of putative errors (inconsistencies or deletions) were verified by Sanger sequencing. Potential open reading frames (ORFs) were identified by use of the GeneMark.hmm (http://opal.biology.gatech.edu/GeneMark) (3), DNAStar (4), and BASys (http://wishart.biology.ualberta.ca/basys/) (5) software packages and were subsequently analyzed manually. The putative functions of the open reading frames (ORFs) were predicted using BLAST (NCBI) (http://www.ncbi.nlm.nih.gov/blast/Blast.cgi) (6, 7) and HHPred (http://toolkit.tuebingen.mpg.de/hhpred). ARAGORN (http://130.235.46.10/ARAGORN/) (8) and tRNAscan-SE (http://lowelab.ucsc.edu/tRNAscan-SE) (9) were used to search for tRNA genes. The PaBG phage replication origin was localized by DNA skew analysis (10).

Negative staining transmission electron microscopy (11) revealed that bacteriophage PaBG belongs to the A1 morphotype of the *Myoviridae* family. The phage particles are composed of a large icosahedral head that is ~136 nm in diameter and a ~220-nm-long contractile tail. The giant *Pseudomonas* phage PaBG has a double-stranded 258,139-bp DNA genome with 55.82% G+C content. The putative functions of at least 94 ORFs out of 308 predicted ORFs in the phage genome can be assigned. Thirteen genes encode the structural components of the virion. Several predicted ORFs were supposed to encode proteins that can be involved in cell wall degradation and lysis of the bacteria: putative sugar binding protein, endolysin, lysozyme (gene 25-like lysozyme of T4 bacteriophage), tail-associated lysozyme (tail spike protein), and muramoylpentapeptide carboxypeptidase. No classic holin was found in the PaBG genome, however.

Other functional ORFs belong mostly to nucleotide metabolism and DNA replication genes. PaBG genes encode the phage's own RNA and DNA polymerases. Transcription is directed both forward (clockwise) for 158 genes (51.3%) and in reverse (counterclockwise) for 150 genes (48.7%) with respect to the replication origin. We identified five genes encoding transport RNAs.

Based on bioinformatics analysis, no sufficient DNA homology between PaBG and other phages with known genome sequences was observed. The closest relative of PaBG is *Pseudomonas putida* phage Lu11 (12, 13). One hundred twenty-five ORFs of these phages are similar on the protein level. We conclude that PaBG phage can be considered phylogenetically distant from other giant *Pseudomonas* phages and that this reflects the diversity within the *Myoviridae* family.

### Nucleotide sequence accession number.

The genome sequence of *Pseudomonas* phage PaBG is available under GenBank accession no. KF147891. The version described in this paper is the first version.
